# Cohen—Croup and Tracheotomy

**Published:** 1874-11

**Authors:** 


CROUP, in its relations to tracheotomy. By J. Solis Cohen, M.D., Lecturer on Laryngoscopy and Diseases of the Throat and Chest, in Jefferson Medical College. Philadelphia: Lindsay and Blakiston, 1874. Octavo, cloth pp. 78. Price $1.00.

Amid all the exigencies of a physician's daily life, we know of none which appeals so strongly to one's sympathies and one's manhood as croup. How distressing is it to see one of these guileless little babes struggling hour after hour for vital air?

With what imploring anguish does the little innocent look up to us for help? How barren do we so often find ourselves of effective remedies? Under such harrowing circumstances how natural the inclination to invoke the aid of the surgeon's knife, that by a stroke we may throw wide the door, and give instant and free entrance to the element of life and hope. It is to assist us in the ready solution of the pressing question of tracheotomy in such emergencies that Dr. Cohen has given us this brochure. It is a careful analysis of a very large number of recorded cases, from which the author draws the following conclusions:

1st. That there are no insuperable contra-indications to tracheotomy in croup. 2d. That the administration of an anæsthetic for the purpose of controlling the child's movements is admissible in performing the operation; but that it should be used with great caution. 3d. That a careful dissection should be made down to the wind-pipe, and hemorrhage be arrested before incising it, whenever there is at all time to do so. 4th. That the incission should be made into the trachea as near the cricoid cartilage as possible, to avoid excessive hemorrhage, and subsequent accidents which might occasion emphysema. 5th. That a dilator should be used, or a piece of the trachea be excised, whenever any difficulty is encountered in introducing the tube. 6th. That the tube should be dispensed with as soon as possible; or altogether if the case will admit of it. 7th. That assiduous attention should be bestowed upon the after-treatment, especially that of the wound; and that a skilled attendant should be within a moment's call for the first twenty-four or forty-eight hours immediately following the operation.

---

ARCHIVES OF DERMATOLOGY. A Quarterly Journal of Skin and Venereal Diseases. Edited by L. Duncan Bulkley, A.M., M.D. Volume 1, No. 1, October, 1874. New York: G. P. Putnam's Sons, 308 Fourth Avenue. Price, $3 per year, in advance; single copy, $1.

This is a handsomely gotten-up pamphlet of 96 pages, to be issued quarterly. It is ably edited, has a most promising array of prominent collaborators, and bids fair to be the standard journal of the country in the specialty of skin and venereal diseases.

---

ERYSIPELAS: ITS TREATMENT WITH SULPHATE OF QUININE. By Y. R. LeMonnier, M.D., visiting Surgeon Charity Hospital, New Orleans, etc. Octavo, pamphlet, pp. 31.

HAND BOOK OF PRACTICE; Employing Concentrated Medicines as prepared by B. Keith & Co., New York. By B. Keith, M.D., 1874. 16mo., cloth, pp. 143.

The design of this little volume is to familiarize the profession with the medical properties and doses of the long list of vegetable active principles, prepared by B. Keith & Co.; and likewise to indicate the diseases in which they are likely to prove beneficial. Those who are beginning the use of these active principles will find the hand-book of Dr. Keith very convenient and valuable.

---

THE CLIMATE AND FEVERS OF THE SOUTHWESTERN, SOUTHERN ATLANTIC AND GULF STATES. By James C. Harris, M.D. Louisville: John P. Morton & Co., 1874. Pamphlet, pp. 18.

